# What normal used to be: Longitudinal adrenal, reproductive, and thyroid hormone profiles from four bowhead whales during a period of Arctic stability

**DOI:** 10.1093/conphys/coag020

**Published:** 2026-04-10

**Authors:** Jennifer Jelincic, Danielle Dillon, Joshua Reed, Matthew C Rogers, Daniela M D de Mello, Alyson Fleming, Nadine Lysiak, Leslie New, C Loren Buck, Kathleen E Hunt

**Affiliations:** George Mason University, Department of Environmental Science and Policy, 4400 University Dr, Fairfax, VA 20030, USA; Marine Mammal Institute, Oregon State University, 2030 SE Marine Science Dr, Newport, OR 97365, USA; Northern Arizona University, Department of Biological Sciences, 617 S Beaver St, Flagstaff, AZ 86011, USA; Anderson Cabot Center for Ocean Life, New England Aquarium, 1 Central Wharf, Boston, MA 02110, USA; Ursinus College, Department of Mathematics, Computer Science and Statistics, 601 E Main St, Collegeville, PA 19426, USA; National Oceanic and Atmospheric Administration, Alaska Fisheries Science Center, 17109 Pt. Lena Loop Rd, Juneau, AK 99801, USA; Northern Arizona University, Department of Biological Sciences, 617 S Beaver St, Flagstaff, AZ 86011, USA; University of São Paulo, Department of Physiology, Institute of Biosciences, São Paulo 05508-090, SP, Brazil; University of Wisconsin–Madison, 122 Science Hall, 550 North Park St, Madison, WI 53706, USA; Anderson Cabot Center for Ocean Life, New England Aquarium, 1 Central Wharf, Boston, MA 02110, USA; Ursinus College, Department of Mathematics, Computer Science and Statistics, 601 E Main St, Collegeville, PA 19426, USA; Northern Arizona University, Department of Biological Sciences, 617 S Beaver St, Flagstaff, AZ 86011, USA; Marine Mammal Institute, Oregon State University, 2030 SE Marine Science Dr, Newport, OR 97365, USA; Smithsonian-Mason School of Conservation, 1500 Remount Rd, Front Royal, VA 22630, USA; George Mason University, Department of Biology, 4400 University Dr, Fairfax, VA 22030, USA

**Keywords:** *Balaena mysticetus*, Biomarkers, Endocrinology, Gestation, Long-term study, Multi-year

## Abstract

Assessment of large whale physiological response and resiliency to environmental change requires information about historical baseline physiological data to compare with current trends. Multi-year studies of historic and modern individual bowhead whales are possible by utilizing baleen, a keratin matrix tissue that captures a time series of physiological data across multiple years. We assessed six hormones in bowhead baleen informative of stress, reproduction, and metabolism: corticosterone, cortisol, progesterone, testosterone, triiodothyronine (T3), and dehydroepiandrosterone (and its sulfated form, collectively DHEA(S)). We also performed assay validations for DHEA(S), as this hormone has not been assessed in keratin. Through examination of longitudinal endocrine profiles of two adult males and two adult females with data from the 1940’s-1960’s, a period of relative Arctic climatic stability, we determined that reproductive cycles could be identified in adult bowhead whale baleen more than sixty years old. Testosterone cycling was observed in males while three putative pregnancies, and a fourth confirmed pregnancy, were observed in females. Strong correlations were observed between DHEA(S) and testosterone in males and pregnant females. Pregnancy displayed the strongest correlations among hormones, indicating that pregnancy is likely a form of physiological stressor that should be controlled for when studying adrenal hormones. However, in non-pregnant females, cortisol was strongly negatively correlated with progesterone and testosterone, indicating that physiological stress may influence ovarian cycling and/or likelihood of future pregnancy. Our findings underscore the importance of utilizing museum specimens to establish historical baselines, as well the value of panels of multiple hormones (representing different physiological axes) when assessing multiple stressors in free-ranging wildlife. Understanding how age, sex, and life-history stage (e.g. pregnancy) influence those hormone patterns is useful for addressing greater conservation-relevant questions, particularly related to environmental change.

## Abbreviations

HPA axis: Hypothalamic–Pituitary–Adrenal axis
HPG axis: Hypothalamic–Pituitary-Gonadal axis
HPT axis: Hypothalamic–Pituitary-Thyroid axis
DHEA(S): Dehydroepiandrosterone (and its sulfated form)
T3: Triiodothyronine
T4: Thyroxine
MeOH: Methanol
EtOH: Ethanol
BGR: Baleen Growth Rate
SIA: Stable Isotope Analysis
pg/mL: Picograms per milliliter
ng/g: Nanograms per gram
mL: Milliliter
mg: Milligram
cm: Centimeter
QA/QC: Quality Assurance/Quality Control
ECWG: East Canada-West Greenland
BCB: Bering-Chukchi-Beaufort
NSB: Non-specific binding
ICC: Intraclass correlation coefficient

## Introduction

Monitoring the physiological response and resiliency of wildlife to environmental change is critical to our understanding of populations and best management practices. Marine mammals are typically long-lived, equating to many opportunities to experience stressors and physiological change in an individual’s lifetime (Booth *et al.,* 2020). Cetaceans (e.g. whales) in particular experience many natural and unique anthropogenic stressors with cumulative effects, which influences vital rates and may ultimately lead to ecosystem-wide impacts. Furthermore, they are expected to be disproportionately impacted by climate change (Simmonds and Isaac, 2007; Burek *et al.,* 2008; Pacifici *et al.,* 2015; National Academies of Sciences, Engineering, and Medicine, 2017; Constable *et al.,* 2022). Thus, it is increasingly important to gain insight into whale life-history strategies (including estimated breeding time, gestation, and inter-calving or interpregnancy intervals), while simultaneously studying their physiological and behavioral response and resiliency to environmental change.

Free-ranging marine mammals, however, are difficult to study and even basic natural history data are limited for some species, typically based only on a relatively small handful of resighting records and necropsies. Physiological metrics are often difficult to assess, due to the inability to collect blood samples from many species. Therefore, studies of the physiology of free-ranging marine mammals have increasingly used alternative samples types such as feces, blubber, saliva, bone, respiratory vapor, and keratinized tissues such as hair, claws, and baleen plates (Palme *et al.,* 2005; Bechshøft *et al.,* 2011; Hunt *et al.,* 2014; Romero and Wingfield, 2015a; Hunt *et al.,* 2017; Burgess *et al.,* 2018; Hunt *et al.,* 2018; Palme, 2019; Crain *et al.,* 2021; Hudson *et al.,* 2021). With these alternative sample types, existing gaps in life-history knowledge may be filled. Some of these sample types have the additional advantage of capturing physiological history across long periods of time, which may enable more detailed examination of the prolonged reproductive cycles characteristic of cetaceans (for example, multiple-year-long inter-calving and/or interpregnancy intervals in females), as well as examination of potential carry-over influences of physiological stress to subsequent future reproductive attempts.

One such sample type is whale baleen, a linear keratin tissue that grows slowly over years. Due to its slow growth rate, baleen can potentially capture the necessary multiple-year time frame to address physiological questions. Baleen is arranged as individual plates growing from the upper jaw as a filter-feeding apparatus. Recent research has demonstrated that baleen studies can provide much needed insight into stress, nutrition, and reproduction in whales via analysis of stable isotopes and steroid and thyroid hormones that are deposited in the baleen as it grows (Schell *et al.,* 1989a, 1989b; Hunt *et al.,* 2014; Hunt *et al.,* 2017a; Fernández Ajó *et al.,* 2018; Fleming *et al.,* 2018; Pomerleau *et al.,* 2018; Rolland *et al.,* 2019; Dedden and Rogers, 2022). Collected postmortem, a single baleen plate provides years of continuous data without the need to recapture individuals.

Bowhead whales (*Balaena mysticetus*) have the longest baleen plates (up to ~ 4 m) and longest life-expectancy of any known mammal (over 200 years) (George, 2009), and thus are of particular interest for studies of response and resiliency to change. Bowheads are an endemic arctic species that occur near the edge of the pack ice (Rugh *et al.,* 2003), migrating southward in winter as pack ice extends, and northward in summers as pack ice retreats. Bowhead baleen has been successfully analyzed for stables isotopes and certain hormones (Schell *et al.,* 1989a, 1989b; Hunt *et al.,* 2014, 2017b). Further, baleen is a robust and stable sample type, with analytes showing no apparent degradation across decades of storage (Schell *et al.,* 1989a, 1989b; Hunt *et al.,* 2014, 2017b, Lowe *et al.,* 2022, Savoca *et al.*, 2024), and archives of bowhead baleen exist that are decades to centuries old. Therefore, this species and sample type may provide retrospective understanding into stress, nutrition, and reproduction for bowheads of historic time periods prior to today’s rapid environmental change.

Bowhead whales have experienced various changes in their arctic environment over the last 150 years and thus provide an interesting example of ecological impacts on organismal physiology. For example, arctic pack ice extent has decreased in recent decades, and an ice-free Arctic Ocean is predicted to occur in summers by approximately 2050, with ice-free winters possible by 2100 (Jahn *et al.,* 2024). Potential impacts on the Arctic marine ecosystem generally and bowhead whales specifically are not known. In contrast, the 1940’s-1960’s is thought to have been a period of environmental stability in arctic and subarctic regions (Gribbin, 1986; Thompson, 1989; Kryzhov and Gorelits, 2015; Bai *et al.,* 2022), and therefore, bowhead baleen from this time period has the potential to provide baseline data that could then be used as a comparison point for studies of modern populations. Specifically, establishing basic endocrine patterns characteristic of typical life-history stages in adult bowheads during a period of Arctic stability may assist us in understanding and predicting responses to cumulative stressors today and in the future.

Certain hormones have been particularly useful for assessing physiological responses to stressors, among them two adrenal glucocorticoids, cortisol and corticosterone. The mammalian hypothalamic–pituitary–adrenal (HPA) axis increases secretion of these hormones in response to a variety of stressful events, and the hormones then help coordinate the vertebrate stress response. Even predictable life-history stages may activate this stress response, such as those life-history stages that require increased energy expenditure, particularly in extreme environments (Jacobs and Wingfield, 2000; McEwen and Wingfield, 2003, 2010; Wingfield and Sapolsky, 2003; Romero and Wingfield, 2015a). Unpredictable stressors tend to be associated with even sharper elevations of these hormones, particularly cortisol in mammals (Romero and Wingfield, 2015b). Historically, much research on the vertebrate stress response focused on just one of these hormones (e.g. cortisol in most mammals, corticosterone in other vertebrates). While the single-metric approach was undeniably useful, it has limitations, such as difficulty distinguishing the type of stressor and duration, which are necessary to quantify the impacts of cumulative stressors (Wingfield and Romero, 2001; Romero and Reed, 2005; Romero and Butler, 2007; Bonier *et al.,* 2009; Busch and Hayward, 2009; Dickens and Romero, 2013; Fefferman and Romero, 2013; Madliger and Love, 2014; Madliger *et al.,* 2016; Palme, 2019; Gormally and Romero, 2020; Romero and Beattie, 2021). Therefore, adding additional hormones and related biomarkers may allow us to differentiate stressors and their impacts.

Another adrenal hormone, dehydroepiandrosterone (DHEA) and its sulfated form (DHEA-S), collectively DHEA(S), may provide a more complete picture of HPA axis function, and recent human and primate research has promoted the incorporation of DHEA(S) in stress and welfare studies. DHEA(S) immunoassays have been validated for some other cetaceans (Robeck *et al.,* 2017; Legacki *et al.,* 2020; Miller *et al.,* 2021; Béland *et al.,* 2023), but not yet in mysticetes (baleen whales). The primary functions of DHEA(S) are not completely understood; DHEA(S) may play a role in the stress response, immune response, memory, anti-aging, the modulation of aggressive or territorial behaviors, and/or circannual timing (Yen, 2001; Soma *et al.,* 2002; Dubrovsky, 2005; Hammer *et al.,* 2005; Takeshita *et al.,* 2013; Edes *et al.,* 2016; Richter *et al.,* 2017; Klinge *et al.,* 2018; Frongia *et al.,* 2020; Whitham *et al.,* 2020; Dutheil *et al.,* 2021; Takeshita, 2022; Béland *et al.,* 2023). DHEA(S) may rise in response to acute stressors and fall during repeated or chronic stress, indicating potential use as an index of allostatic load (Edes *et al.,* 2020; Whitham *et al.,* 2020). Thus, investigation of DHEA(S) in mysticetes, including examination of any positive or negative correlations with other adrenal or reproductive hormones, seems warranted.

Two other steroid hormones, testosterone and progesterone, can provide substantial information to assess the activation of the hypothalamic–pituitary-gonadal (HPG) axis and potential breeding events. For example, annual testosterone cycling has been shown in one population (East Canada-West Greenland (ECWG)) of bowheads (Hunt *et al.,* 2022), with a rise in testosterone concentrations in baleen coincident with the putative breeding season. Occurrence of such testosterone cycles may provide information on age of sexual maturity, regularity of reproductive cycling, and potentially senescence in old individuals. Similarly, in females, a dramatic and prolonged elevation of progesterone in baleen grown during pregnancies (both putative and confirmed with calf sightings) has been confirmed in bowheads and closely-related species (Hunt *et al.,* 2016; Lysiak *et al.,* 2023). Thus, examination of progesterone patterns may allow identification of putative pregnancies as well as calculation of inter-calving and/or interpregnancy intervals, an important cetacean life-history parameter that has profound importance for population reproductive rate and population resilience. Little information currently exists on inter-calving intervals in bowheads; older literature suggests an estimated typical interval of three years (e.g. three years between the birth of one calf and birth of the next), but one recent study in the ECWG population documented longer intervals (Lysiak *et al.,* 2023). As the Bering-Chukchi-Beaufort Sea (BCB) population is considered a stronghold for the species and is the population of focus for this study, the examination of testosterone and progesterone patterns in even a few individuals could provide much-needed information.

Finally, thyroid hormones are important in both stress and reproduction. Thyroid hormones coordinate metabolism and growth in all life-history stages in mammals, are the primary determinants of metabolic rate, and have been shown to change during food limitation, thermal stress, and/or periods of unusual energy expenditure (Mullur *et al.,* 2014; Atkinson *et al.,* 2015; Behringer *et al.,* 2018; Lysiak *et al.,* 2018). The hypothalamic–pituitary-thyroid (HPT) axis controls secretion of the inactive form of thyroid hormone, thyroxine (T4), which is converted by target tissues to the active form of thyroid hormone, triiodothyronine (T3). Prior research on some cetaceans as well as terrestrial wildlife suggests that evaluation of T3, in comparison to the glucocorticoids, can also help discriminate periods of nutritional stress from other types of stressors (Mullur *et al.,* 2014; Atkinson *et al.,* 2015; Behringer *et al.,* 2018).

Evaluated together, this panel of six hormones—cortisol, corticosterone, DHEA(S), testosterone, progesterone, and T3—can potentially be used to examine the function of multiple body processes and axes (HPA, HPT, and HPG) during varying life-history stages, as well as the way in which they may shift in response to environmental factors. In this study, we examined a six-hormone panel for four individual bowhead whale baleen plates (two males and two females). Each plate represents a unique case study for repeated sampling across multiple years. The benefit of repeated samples within each plate is that we can view individual variation across multiple years (i.e. > 50 samples per individual, each assayed for six analytes). As five of the six hormone assays have already been validated for bowhead baleen and confirmed to be detectable, our first objective was to determine if the sixth, DHEA(S), is detectible in baleen, which would be, to our knowledge, the first confirmation of DHEA(S) detectability in a keratin tissue. Our second objective was to compare the longitudinal profiles of all six hormones (corticosterone, cortisol, DHEA(S), progesterone, testosterone, and T3) and evaluate associations between hormones during different reproductive life history stages (e.g. putative pregnancy) during a period of arctic stability. Our final objective was to determine if cycles in reproductive hormones could be identified in adult bowhead baleen that is more than sixty years old. In males, we expected annual testosterone cycles similar to those seen in modern bowheads (Hunt *et al.,* 2022). We expected females to have occasional pronounced elevations in progesterone (e.g. putative pregnancies). As one female was confirmed pregnant at her death (Female 1), we examined the base of her baleen plate to determine the progesterone pattern characteristic of pregnancy and expected similar progesterone patterns during other putative pregnancies. We were particularly interested in variation; we wanted to determine if testosterone cycles appeared regularly and if putative pregnancies and interpregnancy intervals were as variable as those observed in modern specimens from the ECWC population of bowheads (Hunt *et al.,* 2022; Lysiak *et al.,* 2023).

## Materials and Methods

### Study Animals

Four adult bowhead whale baleen plates (two males, two females) were available for this study at the Natural History Museum of Los Angeles County, all of which were originally collected in the 1960’s via Iñupiat subsistence hunting in Utqiaġvik, Alaska. Male 1 (LACM 54505) was collected 1964-May-04 and was 12.8 m body length at time of death, Male 2 (LACM 54805) was collected on 1964-May-03 at greater than 14.94 m, Female 1 (LACM 54486) was collected 1969-May-27 at 13.72 m with a fetus, and Female 2 (LACM 72490) was collected 1965-September-21 at 16.76 m. All four individuals were classed as adults based on body length at death (George *et al.,* 2021, 2024; Tarpley *et al.,* 2021).

### Baleen Plate Preparation

We cleaned both sides of each baleen plate with 70% ethanol (EtOH) three times to remove any surface contamination. We then labelled the baleen plate with cm markers starting at the base (gum end; most proximal point and recently grown baleen) of the plate, which was considered 0 cm, using laboratory tape near the labial side following the natural curvature of the plate. All baleen plates were intact at the proximal (gum) end. Perpendicular to the measuring tape, tape was placed at every other even cm mark on the labial side of the baleen plate to serve as a guide for where to collect the sample. Baleen was abraded using a Dremel rotary tool with a tungsten carbide carving bit and resulting baleen powder was transferred into anti-static bags. Excess powder was removed, and the baleen plate was cleaned with compressed air and disinfected with 70% EtOH in between samples to prevent contamination of adjacent sampling sites. Powder was collected for stable isotope analysis (SIA) every 2 cm, and for hormone assays every 4 cm.

### Stable Isotope Analyses (SIA)

Because evidence suggests bowheads are year-round feeders (Pomerleau *et al.,* 2018) with cyclic patterns in ingested and integrated stable isotopes (Schell *et al.,* 1989a, 1989b) from prey in summer versus winter feeding grounds, we used stable isotope analyses (SIA) to identify the timeline represented by each baleen plate (i.e. number of years of growth and calendar year of each region of the plate, based on date of collection and annual stable isotope cycles). To determine stable isotope values, we weighed approximately 1 mg of baleen powder into tin capsules. Bulk carbon (δ^13^C) and nitrogen (δ^15^N) isotope ratios were measured (in ‰) using a Thermo Flash*Smart* Elemental Analyzer coupled into a Thermo Finnigan DELTA^plus^XP continuous-flow isotope ratio mass spectrometer. Isotope ratio values were calculated using the equation δ_sample_ = [R_sample_/R_standard_—1] * 1000 (^13^C/^12^C or ^15^N/^14^N are the ratios for R_sample_ and R_standard_) (Peterson and Fry, 1987). Laboratory QA/QC controls included homogenized Chinook salmon muscle and purified methionine (NOAA Auke Bay Laboratories). Isotopic reference materials, error, and precision follow previously published protocols (Fernández Ajó *et al.,* 2024).

### Hormone Extraction and Assays

Baleen hormone extraction followed previously validated methods (Hunt *et al.,* 2014, 2017b; Fernández Ajó *et al.,* 2022). 20 mg of each baleen powder sample were weighed (range of 19.8–21.2 mg) and 4 mL 100% HPLC-grade methanol (MeOH) was added to a 13x100 mL borosilicate glass vial. Samples were vortexed for two hours, then centrifuged for 15 minutes at 3000 g. 3.6 mL of the resulting supernatant was recovered from the vials and transferred into a new 12x75 mL vial, dried in a rotary evaporator, and resuspended in 500 μL of Arbor Assays (Ann Arbor, MI, USA) X065 buffer, with results later corrected for the 0.4 mL of supernatant that was not recovered. Vials were then vortexed for 5 minutes, sonicated for 5 minutes, and transferred into 1.5 mL vapor proof o-ring-capped cryovials, frozen overnight, and decanted into a new cryovials to remove any remaining baleen powder particles. The resulting 1:1 dilution (i.e. ‘neat’) was stored at −80°C until ready for immunoassay analysis.

DHEA(S) parallelism was first assessed by comparing the slope of the dose–response curves of hormone standards to that of serial dilutions of a bowhead baleen sample pool, with equal slopes considered ideal. Therefore, to test for parallelism, a pool of bowhead baleen extract was serially diluted in assay buffer to produce eight total dilutions (1:1 to 1:128). The dilutions were assayed as unknowns using the Arbor Assays DHEA-S assay (#K054), and the linear portions of the slopes of the standards and bowhead pool were compared (see Statistical Analyses). Next, DHEA(S) assay accuracy was assessed, also known as a matrix effect test, which tests whether the assay correctly identifies low- and high-dose samples without interference from the sample matrix. Accuracy was assessed by using a 1:1 bowhead baleen extract to spike a full standard curve, comparing assay results to a second standard curve spiked only with assay buffer, followed by the assessment of linearity and slope of expected vs. observed hormone (see Statistical Analyses). Cross reactivity of DHEA-S and DHEA is 100%, therefore, we consider the assay to detect a combination of both forms; we refer to both analytes collectively as DHEA(S). As DHEA is an androgen, other cross reactants for this assay include several related androgens: epiandrosterone (44.5%), androsterone (28.4%), and androstenedione (15.2%). Cross reactivity is less than 0.5% for testosterone and all other tested steroid hormones.

All hormones were quantified with commercially available enzyme immunoassay kits from Arbor Assays validated in bowheads (Hunt *et al.,* 2014, 2017b). Assays were run according to manufacturer protocols, with minor adjustments to standard ranges as follows: Corticosterone (Catalog #K014) was assayed with a standard range of 78–5000 pg/mL, cortisol (#K003) at 25–1600 pg/mL, DHEA-S (#K054) at 96–60000 pg/mL, progesterone (#K025) at 50–3200 pg/mL, testosterone (#K032) at 41–10000 pg/mL, and T3 (#K056) at 39–2500 pg/mL. Cortisol, corticosterone, and T3 samples were assayed at a dilution of 1:2 while DHEA(S), progesterone, and testosterone were assayed at a dilution of 1:4. These dilutions were selected to attain acceptable assay accuracy and precision while also minimizing the volume of rare sample needed.

QA/QC criteria for all assays included standard procedures: each microplate including a full standard curve, NSB (non-specific binding), zero (no hormone), species-specific pools, and lab controls, when available. Intra-assay and inter-assay coefficients of variation (CVs) for all assays were < 10%.

### Statistical Analyses

#### Year estimation and baleen growth rate

To determine which point along the baleen (which cm measurement) equated to which calendar year of growth in each baleen plate, we determined yearly cycles in δ^13^C and δ^15^N using R package *scorepeak* (Ochi, 2019), with window size set to eleven. Peaks in stable isotopes were scored by comparing the midpoint of the window to its neighbors on either side within the length of the window. Once peaks were identified, peak placement was visually assessed for both δ^13^C and δ^15^N to ensure peaks were not missed due to lack of variability in one isotopic value compared to the other. Prior literature suggests that post-nuclear weapons testing, δ^13^C was the most reliable peak detection isotope in the BCB population (Schell *et al.,* 1989b). However, δ^13^C peaks can sometimes be “missing” (e.g. a whale does not migrate as usual, or prey selection is unusual in a given year). Thus, if an instance of a δ^13^C peak-to-peak spacing was identified as being more than 1.5 times the expected baleen growth rate for that individual, δ^15^N and average baleen growth rate were used to identify the existence of a missing peak for that year, which was then manually added to the timeline. Because peaks in δ^13^C have been previously shown to indicate peak winter feeding in BCB bowhead whales (Pomerleau *et al.,* 2018), we assigned peak δ^13^C values a calendar date of the 41^st^ day of the year, which is the average winter midpoint between first detection of bowhead calls (>3 hours) leaving the Bering Sea (October–November) and the last detection of bowhead calls (>3 hours) returning to the Bering Sea (April–May) estimated from acoustic detections in the Bering Strait (Szesciorka and Stafford, 2023). The remaining dates of the year were calculated as:


$$ day=\frac{365}{peak\ to\ peak\ length}\times sampling\ distance $$

where sampling distance is the number of cm between consecutive samples (2 cm). Calendar year was then added to the dataset working back from the recorded year of death.

#### Hormones

DHEA(S) assay parallelism was assessed with an F-test, with a non-significant p-value signifying that the slopes of the standard curve and the serially diluted bowhead baleen pool are not statistically different. Thus, the standard curve can reliably by used to interpolate hormone concentrations. To determine DHEA(S) accuracy, expected hormone concentration was plotted against observed hormone concentration and a linear regression was fit to the data. Acceptable accuracy was defined as r^2^ > 0.95, and a slope within 0.7 and 1.3 (ideal slope = 1.0) (Grotjan and Keel, 1996). Hormone concentrations were interpolated using a four-parameter logistic curve fit and were performed using GraphPad Prism 10.4.1 (Boston, MA, USA). All other analyses were performed in R (R Core Team, 2026). Baseline hormone concentrations were determined using R package *hormLong* (Fanson and Fanson, 2014) and Spearman’s rank correlation values between hormones (Tu **et al*.,* 2025a) were obtained in base R and visualized using R package *corrplot* (Wei and Simko, 2024), and the rank intraclass correlation coefficient (ICC) was calculated using the R package *rankCorr* (Tu *et al.,* 2025b) to assess the contribution of individual effects to the total Spearman rank correlation. The rankCorr estimator can result in negative rank ICC values when there are only two clusters (as is the case here), but the estimates of total, within- and between-cluster correlation have very low bias and good coverage even when this is the case (Tu *et al.,* 2023). Reproductive life-history stages were identified in males by accessing occurrence and inferred season of regularly spaced testosterone peaks. Females were assigned putative pregnancies for those periods of time by prolonged progesterone elevations (e.g. above baseline) separated by low progesterone intervals on either side. Female 1 was pregnant at her death; therefore, this pregnancy was confirmed even without subsequent low progesterone values.

## Results

### Year Estimation and Growth Rate

Stable isotope analyses indicated that each whale had between 12 and 16 continuous years represented in their baleen plates (Supplementary Material Fig. S1), with the largest whales of both sexes having a slower baleen growth rate (BGR), as expected, and therefore more years represented in their baleen plates (Female 2 and Male 2). Morphometric data and BGR average in cm/year and associated calendar years (backdated from collection date) are provided in Table 1. Female 1’s baleen contained 13 putative annual isotope cycles (years), Female 2 had 16, Male 1 had 12, and Male 2 had 14 years.

**Table 1:** Morphometric data (body length in meters (m) and baleen plate length in centimeters (cm) for each individual, followed by calculated baleen growth rate (BGR) in centimeters per year (both average and range for full calendar years only; partial years were omitted). Using the collection date and BGR calculations, the calendar years represented in the baleen plate are also provided

**Individual**	**Body Length (m)**	**Baleen length (cm)**	**BGR (cm/year)**	**Calendar Years**
**Female 1**	13.72	267.97	Average: 19.45 Range: 14–22	1956–1969
**Female 2**	16.76	303.53	Average: 17 Range: 14–22	1949–1965
**Male 1**	12.80	246.38	Average:19.73 Range: 16–22	1952–1964
**Male 2**	>14.94	241.3	Average: 16.77 Range: 16–22	1955–1969

### DHEA(S) Parallelism and Accuracy

**Figure 1:**
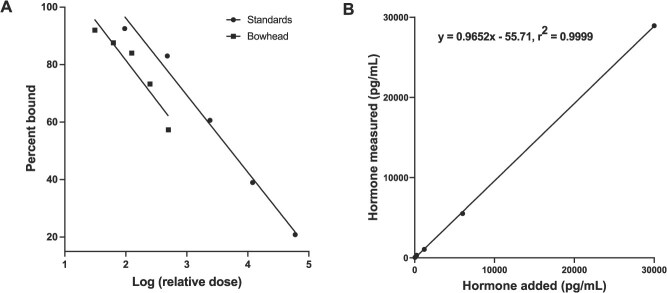
DHEA(S) validation results for pooled baleen extracts. (A) indicates DHEA(S) parallelism of serially diluted samples to standard curve (F = 0.03726, DF = 1,6, p = 0.8533) and (B) shows DHEA(S) accuracy graph with best-fit linear regression line that has a slope near 1.0 (y = 0.9652x—55.71, r^2^ = 0.9999).

The serial diluted baleen extract and pure hormone standards demonstrated acceptable parallelism, i.e. no significant difference in slope between the linear portions of the binding curves (*F*_1,6_ = 0.03726, *p* = 0.8533; Fig. 1A). The best-fit linear regression line for accuracy had a slope acceptably close to 1.0 (0.9652), with an r^2^ of 0.9999 (Fig. 1B).

### Longitudinal Endocrine Profiles and Hormone Associations

**Figure 2:**
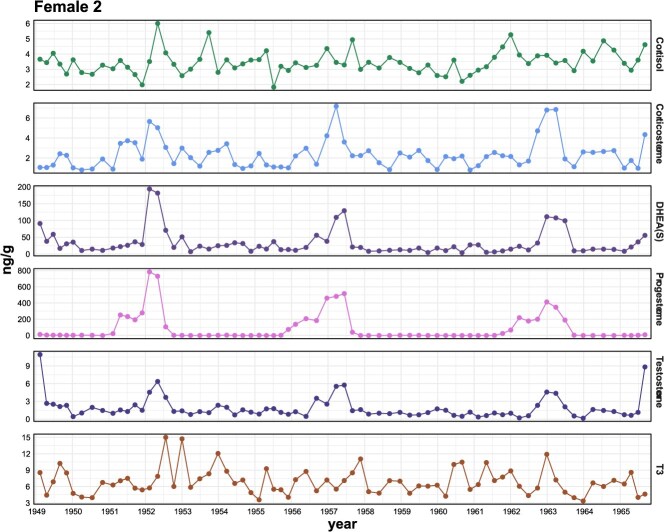
Mature female hormone profile presenting all 6 hormones (cortisol, corticosterone, DHEA(S), progesterone, testosterone, and T3) for bowhead (showing Female 2). The x-axis displays the time-series data (baleen plate cm markers) converted to year using stable isotope data with gray shading every other year. Rises in progesterone indicate putative pregnancies.

The two largest whales, and presumably the oldest (Female 2 and Male 2) (George *et al.,* 2021), had the highest baseline concentrations of DHEA(S) (Table 2). Reproductive hormone profiles varied by individual, as follows: Female 1 had high progesterone at the base of the plate, corresponding with her confirmed pregnancy; however, she had no other putative pregnancies (no notable progesterone elevations) in any prior part of the baleen plate (Supplementary Material Fig. S2). Female 2 had three putative pregnancies in her longitudinal profile (Fig. 2), as defined by pronounced (above baseline) and sustained (>1 year) rises in progesterone.

**Table 2:** Mean (M), range (e.g. minimum and maximum), and baseline (B) concentrations in nanograms per gram (ng/g) for each individual whale, across all life-history stages

**Individual**	**Mean, Range, and Baseline Hormone Concentrations (ng/g)**					
	**Cortisol**	**Corticosterone**	**DHEA(S)**	**Progesterone**	**Testosterone**	**T3**
**Female 1** *n = 64*	M: 0.79 0.04–3.06 B: 0.64	M: 2.60 1.58–4.21 B: 2.47	M: 19.43 3.17–148.93 B: 17.11	M: 39.82 1.48–481.80 B: 6.84	M: 3.36 0.29–7.92 B: 3.14	M: 5.43 2.44–8.79 B: 5.28
**Female 2** *n = 72*	M: 3.46 1.82–6.01 B: 3.27	M: 2.34 0.81–7.2 B: 1.79	M: 33.42 3.93–193.83 B: 15.19	M: 88.92 0.06–785.52 B: 0.06	M: 1.89 0.15–10.92 B: 1.03	M: 6.92 3.37–15.02 B: 6.18
**Male 1** *n = 58*	M: 3.44 1.74–5.1 B:: 3.34	M: 2.15 0.68–6.30 B: 2.00	M: 57.51 8.85–575.99 B: 34.86	M: 0.46 0.06–7.62 B: 0.06	M: 3.37 0.21–14.52 B: 2.68	M: 7.70 2.84–13.48 B: 7.50
**Male 2** *n = 58*	M: 3.39 2.07–5.29 B: 3.24	M: 1.87 0.76–5.04 B: 1.82	M: 61.63 10.49–245.59 B: 50.39	M: 1.64 0.06–35.41 B: 0.06	M: 2.85 0.37–13.11 B: 1.95	M: 7.02 3.34–13.15 B: 5.52

**Figure 3:**
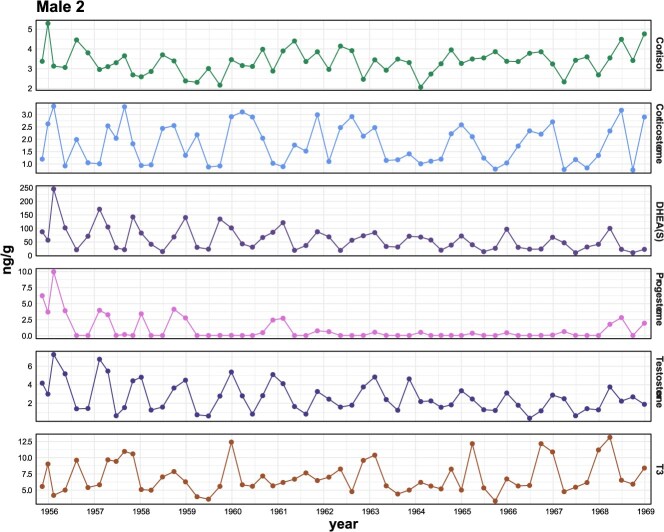
Example mature male hormone profile presenting all 6 hormones (cortisol, corticosterone, DHEA(S), progesterone, testosterone, and T3) for bowhead (showing Male 2). The x-axis displays the time-series data (baleen plate cm markers) converted to year using stable isotope data with gray shading every other year. Note the value nearest the base (gum) of the plate (earliest time period—1969—has been omitted from the graph (see Table 2 range for values).

Male 1 did not show the expected annual testosterone cycling patterns for half of his hormone profile (Supplementary Material Fig. S3), which was a stark contrast to the annual, clear, regular testosterone cycling detected in Male 2 across the full length of his baleen plate (Fig. 3). In the earlier half of Male 1’s testosterone profile, there appeared to be some variation in his testosterone concentrations, however there was no distinct, annual cycle until around 1958, corresponding with a simultaneous rise in baseline T3.

**Figure 4:**
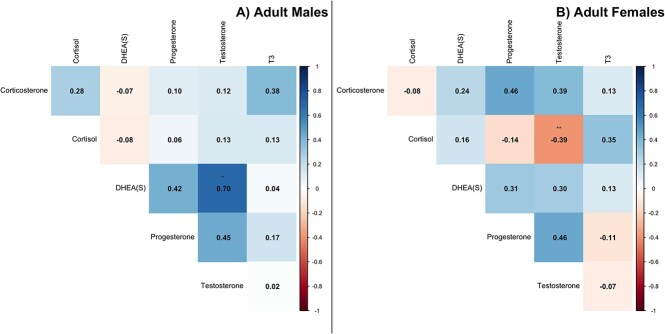
Spearman’s rank correlation plots for corticosterone, cortisol, DHEA(S), progesterone, testosterone, and T3 in adult males (A) and adult females (B.). The darker the blue, the stronger the positive correlation, while the darker the red the stronger the negative correlation. Values close to zero are white. Significant p-values are indicated with stars: *0.05, **0.01, and ***0.001.

Spearman’s rank correlation values indicated a strong and significant positive relationship between DHEA(S) and testosterone concentrations in adult males ($ \rho$ = 0.70, p-value = 0.023; Fig. 4A), and the rank ICC values were effectively zero (DHEA = −0.002, Testosterone = −0.014). While there was an observed positive correlation between progesterone and DHEA(S) ($ \rho$ = 0.42) and testosterone ($ \rho$ = 0.45), these values were not statistically different than zero. Adult females, however, had a significant negative association between testosterone and cortisol (i.e. across the entire plate, combining both putative pregnant and non-pregnant periods; $ \rho$ = −0.39, p-value = 0.004; Fig. 4B), although there is some indication that individual level effects are contributing to this relationship (rank ICC cortisol: 0.731, rank ICC testosterone: 0.26). The other observed positive correlations between progesterone and testosterone ($ \rho$ = 0.46) and progesterone and corticosterone ($ \rho$ = 0.46) were not statistically different from zero.

**Figure 5:**
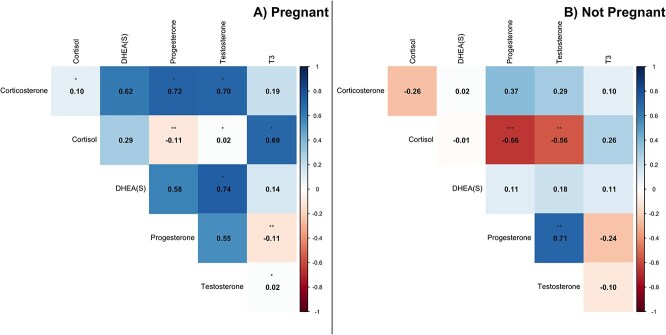
Spearman’s rank correlation plots for corticosterone, cortisol, DHEA(S), progesterone, testosterone, and T3 in pregnant adult females (A) and non-pregnant females (B.). The darker blue, the stronger the positive correlation, while the darker the red, the stronger the negative correlation. Values close to zero are white. Significant p-values are indicated with stars: *0.05, ^**^0.01, and ^***^0.001.

When separating these analyses for females into pregnant versus non-pregnant periods, several strong and statistically significant relationships emerged in adult female baleen hormone concentrations (Fig. 5A and Fig. 5B). During putative pregnancy (i.e. excluding low-progesterone periods), progesterone was strongly and significantly positively correlated to corticosterone ($ \rho$ = 0.72, p-value = 0.019) and a significant, albeit mild, negative association with both cortisol ($ \rho$ = −0.11, p-value = 0.005), and T3 ($ \rho$ = −0.11, p-value = 0.006). Testosterone had a strong, significant positive relationship with corticosterone ($ \rho$ = 0.70, p-value = 0.038) and DHEA(S) ($ \rho$ = 0.74, p-value = 0.027). There was no evidence for an individual-level effect in these correlations, as the rank ICC values were effectively zero for progesterone, corticosterone, and DHEA, while being weak for T3 (0.295). Cortisol had a significant positive relationship with T3 ($ \rho$ = 0.69, p-value = 0.020), although here there was a potential moderate similarly between cortisol values (rank ICC 0.45) from the same whale.

Conversely, when a female was not pregnant (i.e. including only the low-progesterone periods), progesterone and testosterone were strongly positively and significantly correlated ($ \rho$ = 0.71, p-value = 0.004) while there were strong, negative relationships between cortisol and progesterone ($ \rho$ = −0.66, p-value < 0.001), and cortisol and testosterone ($ \rho$ = −0.56, p-value = 0.003). However, there is strong similarity between measurements of corticosterone (rank ICC 0.74) from the same whale, and moderate similarity between measurements of testosterone (rank ICC 0.413) and progesterone (rank ICC 0.613).

## Discussion

Our study demonstrates that a relatively small panel of steroid and thyroid hormones shows great potential for examining patterns in stress and reproduction across time in mysticetes. The four baleen specimens analyzed here each contained continuous endocrine data spanning more than a decade (12–16 years, depending on the individual). While the large number of samples from each individual necessarily limits the number of total individuals, the detailed time series from each individual provide valuable insight into both individual hormone variation present in the species, particularly by life-history stage. The strongest individual ICC effects observed were in nonpregnant females, in which we had life-history stages that could not be identified, which could explain the variation as much as individual whale variation. These four individuals can thus begin to inform our understanding of ‘what normal used to be’ in a past period of arctic stability, after the peak of commercial whaling but prior to many impacts of climate change, and have the potential to serve as a benchmark for future work.

### DHEA(S)

Our study included the first validation of a DHEA(S) assays for any keratin tissue. DHEA(S) was not only readily detectable in bowhead baleen but also provided insight into how the HPA-axis and HPG-axis are both activated during reproduction. Generally, DHEA(S) was more strongly correlated to the reproductive hormones than to either of the other adrenal hormones. Structurally, DHEA(S) is classed as an androgen, though it does not bind well to the androgen receptor, and its physiological role is not thought to be a classic androgen role (Clark and Klinge, 2023). It is primarily produced in the adrenal glands (Baulieu *et al.,* 1965), but also is produced in the gonads (Hindmarsh and Geertsma, 2017), and DHEA(S) can be converted into active sex steroids (Labrie *et al.,* 1995, 1998). Additionally, the DHEA(S) assay used in this study shows cross reactivity to certain androgens (though not to testosterone specifically), most of which have not been evaluated in mysticete whales. Thus, it is possible that our DHEA(S) data may actually reflect as-yet unidentified androgens that might be present in mysticete whale tissues. Indeed, in both males, DHEA(S) data generally paralleled testosterone. In the larger male, we observed regular winter elevations in DHEA(S) that generally parallelled testosterone. In the smaller male, there were only two prominent DHEA(S) peaks, but even in this individual, DHEA(S) appeared to parallel testosterone (i.e. minor DHEA(S) elevations coincident with testosterone peaks). In females, DHEA(S) showed a prominent elevation during each putative pregnancy. As DHEA(S) is also a precursor hormone to certain estrogens (Labrie *et al.,* 1995), DHEA(S) may correlate to estrogens (not measured in this study), which also rise later in pregnancy (Lysiak *et al.,* 2023). Clearly, further research is warranted. Overall, the DHEA(S) assay investigated here shows promise as a proxy of reproduction in both sexes. Additionally, the amplitude of DHEA(S) peaks declined over time in the oldest male and oldest female, and thus DHEA(S) may provide insight into the aging process in bowheads (Labrie *et al.,* 1998).

### Progesterone and Pregnancy

As predicted, both females showed pronounced and prolonged elevations in progesterone that we interpret as pregnancies. Prior studies on modern bowheads and related species confirmed that such progesterone patterns accurately reflect known pregnancies (Hunt *et al.,* 2016; Lysiak *et al.,* 2023). This study illustrates that similar progesterone patterns are readily detectable even in archival specimens of baleen stored at room temperature for over 50 years. In addition, Female 1 provided an additional biological validation as this adult-length female (13.72 m) was documented at necropsy to have a fetus, and indeed progesterone was highly elevated in the baleen growth in the ~ 1.5 years just prior to death. She had no evidence of any other pregnancies during the previous decade of baleen growth, suggesting this may have been her first pregnancy. This was unexpected given that her body length at death of 13.72 m suggests that she was old enough to have experienced at least one prior pregnancy. A recent analysis, however, showed that though the smallest known mature females were 12.6 m in length, the estimated length for a 95% confidence of female sexual maturity is 15.1 m (George *et al.,* 2024), and thus she may only have recently attained sexual maturity. It is worth noting, however, that all specimens included in George *et al.,* 2024 were collected after 1973, and there is a trend of a decreasing length at sexual maturity (i.e. the adult females collected after 1973 were likely smaller than adult females were in the 1940’s-1960’s), consistent with climatic trends (Sheridan and Bickford, 2011). Thus, it is possible that Female 1 was ‘big-for-her-age’ by today’s standards, and that her documented pregnancy may indeed have been her first. This case, though admittedly inconclusive, demonstrates that the multi-year timeframe provided by baleen analysis may enable investigation of the effects of age and body size on sexual maturity, relevant for the modern era in which many whale species are suspected to be smaller in body dimensions than they were in the past.

During putative pregnancies of both females, we observed a biphasic (‘double-peak’) progesterone profile, which is consistent with longitudinal pregnancy profiles of some other mammalian species (Foreman and Garris, 1984; Dörr *et al.,* 1989), including other cetaceans (Hunt *et al.,* 2016; Lysiak *et al.,* 2023; Orbach *et al.,* 2024). In other mammals that show this pattern during pregnancy, the first peak is thought to correspond with initial corpus luteum progesterone secretion after conception, while the second, more pronounced peak later in gestation reflects the shift to placental secretion of progesterone (Harrison and Ridgway, 1971; Duffield *et al.,* 1995; Bergfelt *et al.,* 2011; Robeck *et al.,* 2012, 2016; Orbach *et al.,* 2024). Interestingly, it was only during the second putative pregnancy peak that DHEA(S) rose. Because DHEA(S) is a precursor to estrogens (Labrie *et al.,* 1995, 1998), this hormone may follow a similar pattern to estrogens, particularly estradiol, as previously observed (Robeck *et al.,* 2016; Lysiak *et al.,* 2023). In many mammals, the transition from corpus luteum to placental production of progesterone is a point at which pregnancies can fail, and thus examination of DHEA(S) in conjunction with progesterone might help identify those pregnancies that have succeeded in making this transition versus those that have failed early.

Female 2 was the only individual with full putative pregnancies and thus, the only animal with which we could assess gestation length and interpregnancy intervals. Her estimated gestation length, defined as the full period of progesterone elevation above baseline, ranged from 17–21 months. This is considerably longer than the previously estimated 14 months for this species (Reese *et al.,* 2001; Tarpley *et al.,* 2021). Progesterone elevations in the modern ECWG population of bowheads (Lysiak *et al.,* 2023) also indicate a longer-than-expected gestation length. Other data from North Atlantic right whales and southern right whales, which are also members of the family Balaenidae along with bowheads, suggest that the Balaenidae overall may have a more prolonged gestation, closer to ~ 1.5 years or longer (Hunt *et al.,* 2016; Shuttleworth *et al.,* 2025). In this study, Female 2 had interpregnancy intervals at 2.93 and 3.67 years (i.e. the end of one pregnancy and the start of another pregnancy), slightly longer than previous estimates (George *et al.,* 2024); however, the modern ECWG population experiences more variable and longer inter-calving intervals (birth of one calf to the birth of the next calf; Lysiak *et al.,* 2023). The BCB population (this study), is considered a larger and more stable population, while the ECWG population is smaller and has not been increasing. Thus, these differences in intervals in two populations and time periods, while limited to a small number of case studies, may be reflective of differences in population-level reproductive rates; investigation into the difference between stocks and/or time periods is warranted, including potential links to climatic impacts including food availability and sea ice retreat (Rugh *et al.,* 1992).

### When is the Breeding Season?

In all four putative pregnancies (one in Female 1 and three in Female 2), the rise in progesterone began over winter, which corresponded with the timing of the observed testosterone peaks in the males. Both males experienced the expected testosterone cycles; however, Male 1 did not experience consistent cycles along the length of his baleen plate. Male 1 had a smaller body length and is assumed to be younger (George *et al.,* 2021), so it is possible that this baleen plate captured his sub-adult years and the beginning of his reproductive testosterone cycling. Male 1 did not have pronounced testosterone peaks until roughly 1958 but based on his ~ 6 years of testosterone peaks and the 14 years of testosterone peaks in Male 1, the estimated breeding season (i.e. months when testosterone is elevated), is November through March. A winter breeding season is consistent with recent observations in the ECWG population (Hunt *et al.,* 2022), although breeding behaviors have been observed nearly year-round (Tarpley *et al.,* 2021). It is thought that reproductive hormone cycles are relatively conserved among mysticete whales, most of which have an annual breeding cycle and fast through their migration to low latitude breeding grounds for parturition and lactation (Orbach *et al.,* 2024). Conversely, bowheads stay in high latitude regions even at the southernmost extent of their migration, and feed year-round (Pomerleau *et al.,* 2018). In sum, our data suggest a winter breeding season, corresponding with a much longer gestation length than originally thought. As both these inferences could have substantial implications for population management (e.g. protection of potential breeding grounds can only occur once season and location of breeding is established), more investigation of these issues is warranted.

### Stress and Reproduction

The negative relationship between cortisol and both progesterone and testosterone in non-pregnant females suggests that elevated cortisol may play an inhibitory role in female reproductive readiness and the likelihood of initiating a new pregnancy. Once a pregnancy is underway, however, cortisol did not have any statistically significant associations with these sex hormones. Instead, corticosterone emerged as the key glucocorticoid associated with reproductive hormones during pregnancy, when it was significantly and positively associated with both progesterone and testosterone. Testosterone was also significantly associated with DHEA(S). Generally, the HPA-axis appears activated during pregnancy, particularly later in gestation (as maintaining pregnancy requires the initial suppression of the stress and immune response) (Thomson, 2013; Robeck *et al.,* 2017; Steinman and Robeck, 2021; Lowe *et al.,* 2021b; Orbach *et al.,* 2024).

Conversely, males did not have a statistically significant relationship between their glucocorticoids and other hormones. It is possible the temporal resolution of baleen prevents detectability of any reproduction-related acute stress that adult males might presumably experience during their testosterone peak (i.e. mating itself is often ‘stressful’ to some degree), though we did not examine this further in this study. Overall, our study demonstrates the value of measuring both glucocorticoids (cortisol and corticosterone), as they had quite different associations with reproduction. Prior research in other mammals has suggested that these two glucocorticoids may provide differing information on acute and chronic stress (Armario *et al.,* 1981; Koren *et al.,* 2012; Xu *et al.,* 2025), and thus together they may offer unique perspectives into reproductive stress, particularly in females—both prior to gestation (cortisol) and in maintaining the pregnancy (corticosterone).

### T3

T3 was reliably detectable and each individual exhibited variation in T3 across the length of their baleen plate. Both thyroid stimulating hormone (TSH) and T4 exhibit seasonal patterns in longitudinal hormone profiles in another large mammal (Brown *et al.,* 2007), and have been shown to change due to nutritional and thermal stress, as well as unusual energy expenditure and reproductive seasons (Mullur *et al.,* 2014; Atkinson *et al.,* 2015; Behringer *et al.,* 2018). As a result, we expected that there would be detectable patterns in bowhead T3 data across time. If, for example, T3 plays a role in modifying energy stores in these capital breeders, we expected to see repeated patterns prior to the winter breeding season in males and prior to pregnancy in females. For the most part, such patterns were not observed. However, there were some indications that T3 may increase when an individual attains sexual maturity: in Male 2, we saw an apparent shift to a new increased T3 baseline after the start of his testosterone cycling and likewise Female 2 appeared to have a decreased baseline T3 concentration prior to her first pregnancy. Clearly, more individuals will be necessary to confirm whether such patterns are widespread in subadult whales approaching maturity. Finally, T3 showed relatively few correlations with any of the steroid hormones, limited largely to a significant positive correlation between T3 and cortisol in pregnant females, and a slight negative association in non-pregnant periods. These findings, while tentative, may reflect different types of physiological stress (e.g. nutritional stress in non-pregnant periods, reproduction-associated stress during pregnancy). While these patterns were subtle, quantification of T3 may still be useful in future studies, particularly for the examination of sexual maturity, for comparison of reproductive versus non-reproductive stressors in adulthood, and for comparisons to modern populations as the arctic climate warms. To provide a better picture of HPT function, other hormones such as T4 and reverse T3 may also be useful in future studies.

### Study Limitations

Though baleen provides a useful sample type in longitudinal physiological studies in mysticetes, there are still some limitations and uncertainties inherent to baleen endocrine analysis. For example, the temporal resolution of any given sample point on the thickness of the baleen plate may vary based on sampling technique (especially sampling depth) and the baleen plate itself, since the innermost portion of the baleen plate (the medulla) is believed to be temporally offset from the outer portion (the cortex) (Rita *et al.,* 2019). That said, the clear rise and fall of stable isotopes and testosterone cycles, and the clear beginning and ends of pregnancies as defined by rises in progesterone, combined with similar patterns observed in baleen of other species, indicate the temporal resolution is likely no worse than (i.e. no broader than) ~ 1–2 months, which should be sufficient to detect seasonal changes accurately. Further, the brief cortisol spikes in baleen of other species corresponding with documented acute-stress events suggest temporal resolution of a given point may be closer to 1 month (Hunt *et al.,* 2017a; Lowe *et al.,* 2021a). It is also possible that the base of the plate (newest growth) may have different hormone extraction efficiency than the tip of the plate, which by its nature, was older and had been eroding in seawater for over a decade longer than the base. Finally, some hormone degradation might occur with the passage of time, which could potentially affect patterns across the length of one specimen, or contribute to trends apparent across multiple specimens. Generally, however, steroid hormones are highly resistant to degradation when in dry environments or within dry tissues (e.g. keratin matrix) due to their robust molecular structure (Miller, 1988), as evidenced by steroid hormone detectability of millennia-old specimens of vertebrate keratin tissues (e.g. archeological human hair; > 10 000 year-old wooly mammoth hair (Webb *et al.,* 2015; Koren *et al.,* 2018; Tisdale *et al.,* 2019; Kellner *et al.,* 2022)). Though we cannot rule out the occurrence of some degradation or alterations in extraction efficiency, consistent hormone concentrations observed across the length of the plates (i.e. from base to tip) indicate that any such effects are likely minor.

Another area of uncertainty in our study is the reliability of using stable isotope cycles to determine year of baleen growth. It is possible, for example, that the occasional apparent cases of a ‘missing,’ inferred isotopic cycle might actually be a case of a dramatic change in baleen growth rate (e.g. what we assumed was two years of baleen growth may have actually been one year). Our baleen growth rate and year determination methods were chosen because our interest in individual variation and specifically, response to environmental change. Previous studies have assumed fairly consistent baleen growth rates in adult whales (Lubetkin *et al.,* 2008, 2012; Busquets-Vass *et al.,* 2017; Hunt *et al.,* 2022; Smith *et al.,* 2024), and we did see even in a period of relative arctic stability, adults had slight variation in their baleen growth per year. Currently, we do not have a way to biologically validate whether any such baleen growth rate variation might occur to an extent that could cause misidentification of years, nor is it clear whether certain environmental factors might in fact cause changes in baleen growth rate (e.g. food availability). It is worth noting that our method for estimating years from isotope cycles produced a good match with yearly testosterone cycling, with only a single testosterone peak detected in each estimated year with consistency in the seasonality of the peak. It is also worth noting that our estimate for peak winter carbon date is derived from modern-day data on the timing of bowhead vocalizations along their migration route between 2009 and 2021, however, it is likely that bowhead migration timing may shift due to climatic effects (Szesciorka and Stafford, 2023; Szesciorka *et al.,* 2024).

While for the most part, we saw no variation between the individuals, that varied for key hormones. This study has an inherent small sample size, and we assumed based on body length, and observed in longitudinal hormone concentrations, that these individuals might have different life-history stages, so we are cautious regarding overarching conclusions.

## Conclusions

Even with the above caveats, it is clear that a relatively small panel of hormones in a small number of individuals can provide useful insight into stress and reproduction in historic samples from mysticetes. In sum, our study provides the first look at detailed individual patterns in stress and reproductive physiology across decade-long timeframes in individual bowhead whales during a period of arctic stability. The profiles presented here are indeed a first step in examining bowheads of the past; future research can add more individuals, more samples, and more pregnancies from the same time period, eventually attaining greater confidence about population averages for baseline hormone concentrations during pregnancy, lactation, and non-reproductive intervals for this species. Ultimately, longitudinal endocrine profiles across the more-than-a-decade timeframe of baleen promotes greater understanding of the normal variation within and among individuals across time. Our hope is that by providing information on stress and reproduction patterns in the past, future research can compare these past baselines to present and future populations, to more accurately understand and predict population-level consequences of a changing Arctic.

## Supplementary Material

Web_Material_coag020

## Data Availability

The data underlying this article will be shared on reasonable request to the corresponding author.
